# Mechanical Properties of Titanium Nitride Nanocomposites Produced by Chemical Precursor Synthesis Followed by High-P,T Treatment

**DOI:** 10.3390/ma4101747

**Published:** 2011-10-06

**Authors:** Edward Bailey, Nicole M. T. Ray, Andrew L. Hector, Peter Crozier, William T. Petuskey, Paul F. McMillan

**Affiliations:** 1 Christopher Ingold Laboratory, Department of Chemistry and Materials Chemistry Centre, University College London, 20 Gordon St., London WC1H 0AJ, UK; E-Mail: edward.bailey@ucl.ac.uk; 2 School for Engineering of Matter, Transport and Energy, Arizona State University, Tempe, AZ 85287, USA; E-Mails: nmthomp2@asu.edu (N.M.T.); crozier@asu.edu (P.C.); wpetuskey@asu.edu (W.T.P.); 3 School of Chemistry, University of Southampton, Highfield, Southampton SO17 1BJ, UK; E-Mail: a.l.hector@soton.ac.uk; 4 Department of Chemistry and Biochemistry, Arizona State University, Tempe, AZ 85287, USA

**Keywords:** metal nitrides, Ti_3_N_4_, synthesis, high pressure, microhardness, nanoindentation, nanocomposite materials

## Abstract

We investigated the high-P,T annealing and mechanical properties of nanocomposite materials with a highly nitrided bulk composition close to Ti_3_N_4_. Amorphous solids were precipitated from solution by ammonolysis of metal dialkylamide precursors followed by heating at 400–700 °C in flowing NH_3_ to produce reddish-brown amorphous/nanocrystalline materials. The precursors were then densified at 2 GPa and 200–700 °C to form monolithic ceramics. There was no evidence for N_2_ loss during the high-P,T treatment. Micro- and nanoindentation experiments indicate hardness values between 4–20 GPa for loads ranging between 0.005–3 N. Young's modulus values were measured to lie in the range 200–650 GPa. Palmqvist cracks determined from microindentation experiments indicate fracture toughness values between 2–4 MPa·m^1/2^ similar to Si_3_N_4_, SiC and Al_2_O_3_. Significant variations in the hardness may be associated with the distribution of amorphous/crystalline regions and the very fine grained nature (~3 nm grain sizes) of the crystalline component in these materials.

## 1. Introduction

Transition metal nitrides form a wide range of refractory materials. Because of their high hardness and mechanical strength they are used as abrasives and high-T structural components and they also provide protective coatings for metals and ceramics, including biocompatible surgical tools and implants [1,2,3]. They usually form metallic compounds with compositions at or below the mononitride stoichiometry (e.g., NbN, MoN, Fe_2_N, TiN_1−x_). However, in a few cases (Ta_3_N_5_, Zr_3_N_4_, Hf_3_N_4_) higher nitrides are known to form semiconducting materials in which the metal ions exhibit the maximum valencies found among corresponding oxides (*i.e.*, Ta^V^; Zr^IV^, Hf^IV^). Other high valency nitrides including Ti_3_N_4_ have also been predicted theoretically [4,5,6,7,8,9]. An amorphous variety of Ti_3_N_4_ was reported to form via ammonolysis of titanium dialkylamides (R = NMe_2_, NEt_2_) followed by thermal treatment of the products in an inert atmosphere [10,11]. We found that that procedure led to products containing significant C and H [12]. However, nearly pure nitride materials with close to the expected Ti_3_N_4_ composition could be obtained by annealing in NH_3_ (Table 1). A similar approach was recently applied to obtain nanocrystalline Hf_3_N_4_ and Zr_3_N_4_ [13]. In our previous work we used X-ray absorption spectroscopy and EXAFS analysis to indicate a local structure based on cubic TiN but with greatly reduced (~40–50%) occupancy of the cation and anion sublattices [12]. Such materials could form via intermediate “ladder” structures as found during ammonolysis of organometallic precursors to produce TaN [14]. Our TEM study of amorphous Ti_3_N_4_ indicated the presence of 8–12 nm nanoparticles of crystalline TiN_x_ embedded within the amorphous matrix [12]. That was an interesting result because analogous amorphous/nanocrystalline TiN/Si_3_N_4_ nanocomposites are reported to possess high hardness [15,16,17,18,19,20]. Here we applied high pressure-high temperature (HPT) techniques to anneal the amorphous/crystalline Ti_3_N_4_ powders and produce dense nitride ceramics to study their mechanical properties.

**Table 1:** Bulk chemical compositions of N, C, H contents of amorphous Ti_3_N_4_ materials prepared in this study and used as precursors for high-P,T annealing experiments. The compounds were produced by precipitation from THF solution following ammonolysis of dialkylamide metal precursors followed by treating at 350–400 °C in flowing NH_3_.

Element wt%	C	H	N
**a-Ti_3_N_4_ precursor**			
% Theoretical	-	-	28.05
% Found 1	3.39	0.12	26.25
% Found 2	3.82	0.93	27.38
% Found 3	3.97	0.60	26.48
% Found 4	4.34	0.60	27.14

Experiments carried out under extreme HPT conditions (P > 10–20 GPa) are being applied to explore formation of various new nitrides and crystalline polymorphs [21,22,23,24,25,26,27,28,29,30,31]. Kroll predicted the relative energetics of crystalline polymorphs of Ti_3_N_4_ using *ab initio* techniques [32]. Beginning with a defect NaCl lattice analogous to our structural model for a-Ti_3_N_4_ [12] he observed transitions to spinel, cubic Th_3_P_4_ or orthorhombic Zr_3_N_4_ structured phases occurring in the P = 10–30 GPa range [7,32]. We recently obtained preliminary results after laser heating a-Ti_3_N_4_ in a diamond anvil cell to P > 17–20 GPa and T~1000 °C and found the appearance of new Raman [33] and X-ray diffraction peaks (A. Salamat, P.F. McMillan, A. Hector *et al.*, in prep [34]). In a lower P range (200–800 MPa) hot isostatic pressing was used to produce dense nitride materials including high hardness composites [18]. Here we combined the chemical precursor approach with high-P,T treatment in an intermediate pressure range (P = 2 GPa) to sinter the amorphous/nanocrystalline powders and produce bulk nanocomposite materials to study their mechanical properties.

## 2. Experimental Procedures

Precursors for our high-P,T experiments were produced by reacting metal dialkylamides (Ti(NMe_2_)_4_, Ti(NEt_2_)_4_: Epichem 99.999%) with liquid NH_3_ (Air Products anhydrous grade, distilled from Na/NH_3_ solution). Metal amide (1 g) was dissolved in THF (20 cm^3^, distilled from Na/benzophenone) and NH_3_ (20 cm^3^) was introduced into the mixture. Precipitation occurred during initial NH_3_ addition and as the mixture warmed with ammonia evaporation. The precipitate was collected by evaporation of the solvent, then dried *in vacuo* and heated to 200–500 °C under flowing NH_3_ to form orange-brown X-ray amorphous solids. Thermogravimetric (TGA) analysis (Mettler Toledo TGA851) produced weight loss profiles similar to those described previously [10,12]. Final N, C, H contents were determined by bulk combustion analysis (Table 1). The composition was found to be nearly equal to Ti_3_N_4_ with only a small contribution remaining from C and H components. We attempted to examine the compositions of the samples after high-P,T treatment using SEM or electron microprobe analysis (JEOL JXA 86,000; 10 kV voltage). The Ti:N ratio could not be determined due to strong overlap between the Ti L and N K X-ray emission peaks. We also attempted TEM EELS experiments but the results were contaminated with oxygen that was introduced during sample preparation. However, some O component might also have become incorporated during sample transfer or the high-P,T annealing processes. Although all samples were stored and loaded into capsules in a glove box environment they might have briefly been exposed to air/moisture during their placement into the piston cylinder apparatus. We expect that the high-P,T environment inside the piston cylinder where the sample was surrounded by a BN capsule and graphite furnace was reducing and N-rich. In the present study we assumed that the bulk compositions remained close to the Ti_3_N_4_ stoichiometry. No evidence for bubbles in any of the samples indicating N_2_ loss was observed following the high-P,T experiments.

**Figure 1:**
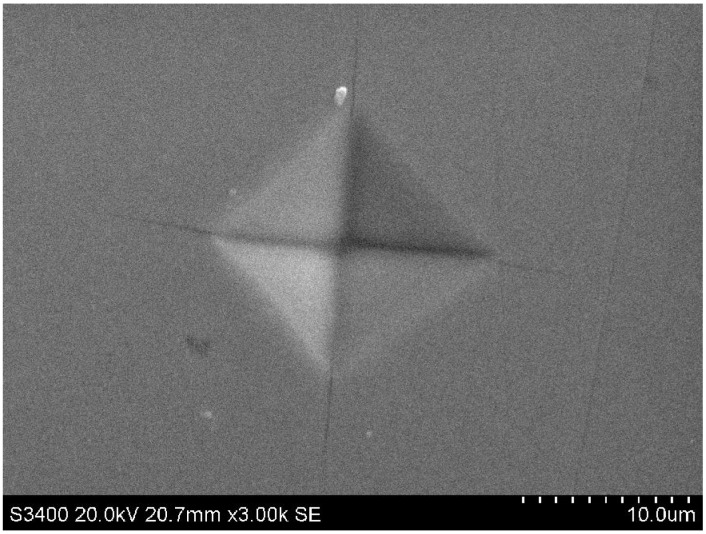
SEM image of sample EB 700 prepared at 2 GPa, 700 °C for 0.5 h. The image shows an indentation produced by a 105 g (1.03 N) load. Cracks emanating from the indentation corners are clearly visible in the Figure indicating brittle behavior. There is also slight bulging along the indentation edges.

High pressure experiments were carried out using a non end-loaded piston cylinder device (Depths of the Earth Co., Cave Creek, AZ, USA) at 2 GPa and T = 200–700 °C for times ranging between 0.5–4 h. One sample was taken to high T at pressure and quenched immediately to provide a “0 hr” test material (Table 2). Samples were pressed into cylindrical pellets (~100 mg) and loaded into h-BN capsules under inert atmospheres (Ar or N_2_) in a dry box. The capsule material is stable under the experimental conditions (melting point 2973 °C) and no reaction with the sample to form phases such as TiB_2_ was observed. Hardness measurements were performed on polished portions of the sample interior. The recovered capsules were mounted in epoxy and cut using a diamond saw and polished for microhardness measurements, then coated with Au for SEM analysis of the indentations (Figure 1). The Au coating was removed later for nanoindentation studies of the same samples.

**Table 2:** Summary of Vickers hardness (*H_V_*) measurements from microindentation experiments on Ti_3_N_4_ samples annealed at 2 GPa. The applied loads ranged between 0.3–3 N.

Sample	T (˚C)	t (h)	Number of measurements	Hardness (GPa)		
				Maximum	Minimum	Mean (1σ) *
EB741	700	4	7	4.8	2.8	4.0 (0.7)
EB746	700	2	12	20.8	10.6	13.3 (3.1)
EB747	700	1	22	18.2	6.1	11.0 (3.0)
EB700	700	0.5	22	14.0	5.2	9.4 (2.5)
EB732	600	4	7	8.3	5.8	7.2 (0.7)
EB743	600	2	17	8.0	3.9	6.2 (1.7)
EB733	600	1	4	6.8	4.6	5.6 (0.9)
EB734	600	0.5	4	6.1	4.0	4.9 (0.9)
EB744	600	0	7	7.0	5.2	6.1 (0.5)
EB699	500	3	6	12.7	6.7	8.0 (2.3)

* Numbers represent mean values of measurements with 1σ values in parentheses. To convert hardness values to conventional non-SI units (kgf/mm^2^) multiply GPa values by 101.97.

**Figure 2:**
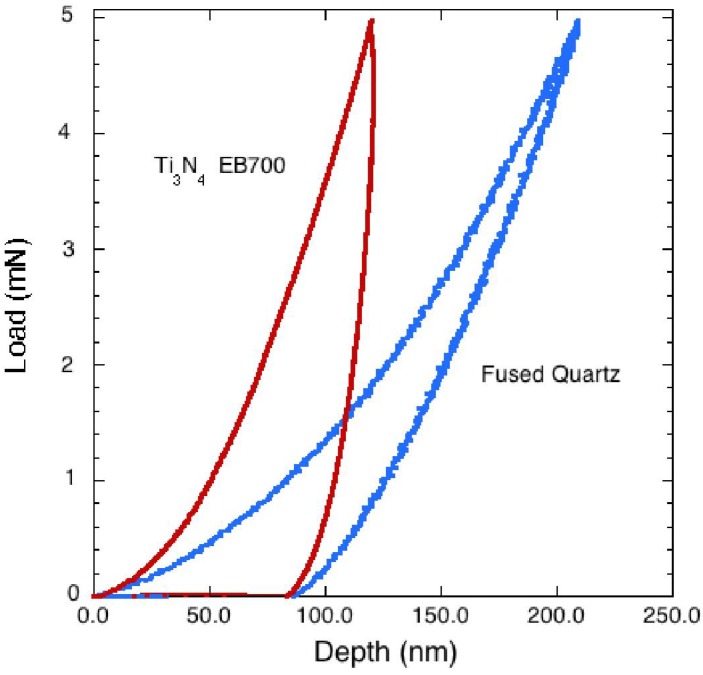
Typical load-depth trace obtained during nanoindentation for sample EB700 up to 5 mN compared with a fused quartz standard. The amorphous/nanocrystalline Ti_3_N_4_ sample exhibits significantly more stiffness while deforming, however, its residual indentation depth on recovery is nearly the same as that of the quartz glass standard.

Microhardness (*H_V_*) measurements were carried out using a Leitz miniload 2 microindenter with a diamond Vickers point and loads applied using 30–2005 g weights to achieve 0.3–19 N. The highest loads resulted in sample failure and hardness measurements reported here were for loads up to 3 N. Indentation areas were measured by both optical microscopy and SEM (Hitachi S-3400 N) techniques. Imaging effects due to pile up at the indentation rims combined with differences in resolution between the two techniques resulted in differences in hardness values. Here we report only results obtained from SEM examination of the indents (Figure 1). Nanoindentation studies were used to obtain complementary hardness data as well as information on Young's modulus (*E*). Data were obtained using a Hysitron Inc. Triboscope nanomechanical tester based on a force-displacement transducer coupled to a multi-mode scanning probe microscope (Digital Instruments Nanoscope Multimode SPM). This was operated in scanning tunneling microscopy (STM) mode for pre- and post-indent imaging. A standard Berkovich diamond tip was used to apply loads ranging between 1–10 mN. The projected contact area of the indent was calibrated as a function of penetration depth using a fused quartz standard (Figure 2).

X-ray diffraction studies of the amorphous precursors and pressure-annealed materials were carried out UCL and Southampton using Cu K_α_ radiation. A spectrum of the amorphous/nanocrystalline precursor material was also obtained at beamline I15 of the Diamond synchrotron using λ = 0.442 Å incident radiation. For TEM measurements, samples previously used for nanoindentation studies were removed from their epoxy mount and lightly ground in acetone then transferred to Cu grids. Experiments were carried out using a JEOL 4000EX instrument operating at 400 kV with a point-to-point resolution of 0.17 nm. Images were recorded on photographic plates and subsequently scanned and processed using Digitalmicrograph software (Gatan, Inc., Pleasanton, CA, USA).

## 3. Results and Discussion

A typical SEM image of a microhardness indent in one of our amorphous/nanocrystalline Ti_3_N_4_ samples prepared using the chemical precursor method followed by high-P,T annealing is shown in Figure 1. The indent is well defined at its edges and is square in shape, comprising 17 µm diagonals, and is similar to indentation figures normally expected for somewhat ductile materials, including a small amount of bulging observed at the indent faces. On the other hand, cracks emanating from the corners indicate brittle behavior. This apparently contradictory combination of properties likely results from the fine grained nanocomposite nature of the sample with ~3 nm crystals coexisting with amorphous domains, as indicated by our TEM and X-ray results. This texture and its likely effects on the mechanical properties are discussed below.

**Figure 3:**
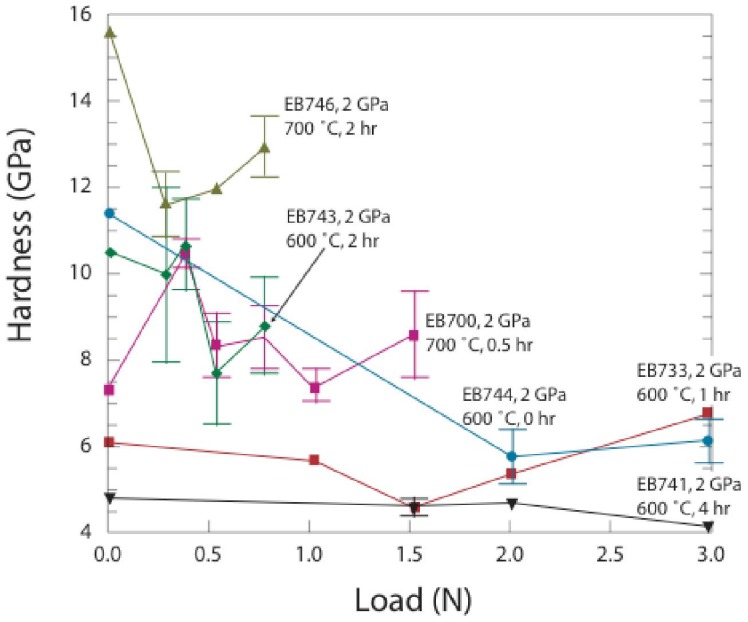
Compilation of hardness values for Ti_3_N_4_ nanocomposite ceramics obtained from microindentation measurements with loads ranging between 0.3–3 N. Samples were treated at 2 GPa and 500–700 °C for times between 0–4 h. Here, we also include averaged values obtained from nanoindentation measurements (Figure 3) at loads < 0.01 N as points at “zero” load. Error bars represent ±1 σ of data obtained at each load.

The microhardness results obtained from measurements of the indent dimensions are summarized in Table 2. Typical values ranged between *H_V_* = 5–17 GPa for applied loads between 0.3–3 N with a generally decreasing trend towards higher loads (Figure 3). No simple relationship between the hardness and P-T-time (*t*) treatment conditions could be determined. The highest hardness values (12–17 GPa) were recorded for one sample treated at 2 GPa and 700 °C for 2 h (EB746), and the lowest (~5 GPa) were observed for EB741 treated at 600 °C for 4 h. Our results indicate lower hardness values than recorded previously for metal nitride materials with compositions close to the mononitride stoichiometry. Sintered polycrystalline TiN with 91–95% theoretical density has a microhardness near 20 GPa [35]. Nanohardness values between 21–22 GPa were quoted for bulk and thin film TiNx materials with x = 0.76–1.0 [36]. Chen *et al.* reported microindentation results for single crystalline NbN, HfN and ZrN, along with elastic constants determined from ab initio theory and X-ray or neutron scattering measurements at high pressure [37]. The Vickers hardness achieved values near 18–20 GPa for HfN and ZrN with ~0.5 N loads, that decreased to 12–18 GPa for maximum loads near 10 N.

We then carried out nanoindentation studies on the pressure-densified Ti_x_N_y_ samples in order to further study their resistance to deformation and obtain complementary information on elastic properties [38]. Hardness (*H_nano_*) was calculated from the maximum load, *P_max_*, and its associated projected contact area, *A_p_*:

(1) $$H_{nano}=\frac{P_{\text{max}}}{A_{P}}$$

**Figure 4:**
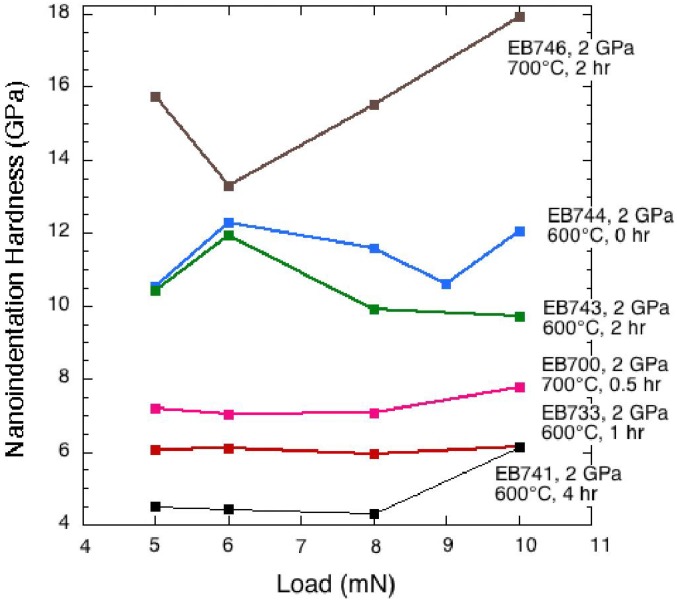
Nanoindentation results for hardness of Ti_3_N_4_ amorphous/crystalline nanocomposite samples for loads ranging between 5–10 mN.

Nanoindentation hardness values can be higher than those determined by micro-indentation [38]. However, the average values of our nanohardness determinations obtained as a function of applied loads ranging between 5–10 mN were consistent with the microindentation values extrapolated to low applied load (Figure 3). Details of the nanohardness results are shown in Figure 4 and Table 3. The maximum nanohardness values ranged up to between 13–18 GPa.

**Table 3:** Summary of nanoindentation hardness measurements for selected Ti_3_N_4_ samples annealed at 2 GPa.

Sample	T (˚C)	t (h)	Load (mN)	Hardness (GPa)	
					Mean *
EB741	700	4	5	4.49	
			6	4.44	
			8	4.32	
			10	6.14	
					4.85 (0.86)
EB746	700	2	5	15.75	
			6	13.29	
			8	15.53	
			10	17.91	
					15.62 (1.89)
EB700	700	0.5	5	7.19	
			6	7.03	
			8	7.07	
			10	7.79	
					7.27 (0.35)
EB732	600	4	5	15.28	
			6	37.6	
			8	25.88	
			10	27.31	
					26.52 (9.13)
EB743	600	2	5	10.42	
			6	11.95	
			8	9.92	
			10	9.71	
					10.50 (1.01)
EB733	600	1	5	6.08	
			6	6.11	
			8	5.96	
			10	6.14	
					6.07 (0.08)
EB744	600	0	5	10.52	
			6	12.27	
			8	11.6	
			9	10.6	
			10	12.06	
					11.41 (0.81)

* Numbers in parentheses represent 1s values from 7–10 data sets.

Young’s modulus values (*E*) were obtained from the measured specimen values (*E_s_*) using the initial slope of the unloading side of the load-displacement cycle (Figure 2) and the diamond indenter (*E_i_*) following the general elastic compliance equation:

(2) $$\frac{1}{E}=\frac{\left(1-\nu_{s}^{2}\right)}{E_{s}}+\frac{\left(1-\nu_{i}^{2}\right)}{E_{i}}$$

Here *ν_s_* and *ν_i_* are Poisson’s ratios for the sample and indenter, respectively. Standard parameters for the diamond indenter are *E_i_* = 1,140 GPa and *ν_i_* = 0.07. We assumed *ν_s_* = 0.3. The resulting *E* values ranged between ~200–650 GPa (Table 3). These cover the range reported in the literature for polycrystalline TiN_x_ samples, depending on the mode of preparation and the measurement method. *E* = 500 GPa has been suggested as a representative value [39,40]. The stiffer materials tended to be those that were annealed at higher T and for longer periods of time. We believe that these results indicate the effects of the microstructure evolution on the sample properties.

Fracture toughness values (*K_lc_*) were estimated from the crack lengths observed in the microhardness studies. The crack dimensions (*a,c*) relative to the microindentation diagonals (*d*) were used to determine the best choice of equation to estimate the fracture toughness, using Palmqvist *vs.* half penny geometries [41,42]. Most cracks were found to satisfy Palmqvist criteria [43]. However, Niihara *et al.* found equally valid results using half penny and Palmqvist models [44,45]. The fracture toughness *K_lc_* (MPa·m^1/2^) was estimated following two alternative models for Palmqvist crack propagation [43]:

(3) Klc(1)=0.009(EH)25ld4c

*vs.*

(4) Klc(2)=0.015(ac)12(EH)23l(d2+c)32

For comparison, we also calculated fracture toughness using the half penny crack model (Table 4 and Table 5) [41,42]:

(5) Kc(3)=0.033(EH)25l(a+c)32

For three samples where we had overlapping information between micro- and nano-indentation results we used the hardness and modulus values directly determined by the nanoindentation experiments (Table 4). However, we also had a much larger data set available from the overall set of crack measurements available from our SEM studies of samples subjected to microindentation. In that case we used the hardness values obtained from microindentation studies, and assumed a representative Young’s modulus value of *E* = 500 GPa that was indicated to be a useful average for TiN_x_ samples [39,40] (Table 5). The fracture toughnesses determined in this study lie within the range 0.8–8 MPa·m^1/2^ (Table 4) or 2–10 MPa·m^1/2^ (Table 5). These span the ranges reported for ceramics including soda-lime-silica glasses (0.3–0.8 MP·m^1/2^) or crystalline materials such as SiC, Si_3_N_4_ or Al_2_O_3_ (3–4 MPa·m^1/2^) [41,46].

**Table 4:** Fracture toughness determinations of selected Ti_3_N_4_ samples annealed at 2 GPa. Hardnesses and Young's moduli, *E*, were obtained from nanoindentation measurements on the same samples. Cracks used are from microindentations. Models (1) and (2) assume Palmqvist cracks, and model (3) assumes halfpenny cracks (see text).

Sample #s	T (°C)	t (h)	E (GPa)	Number of measurements	*K_lc_*(1) MPa·m^1/2^	*K_lc_*(2) MPa·m^1/2^	*K_lc_*(3) MPa·m^1/2^
					Mean **	Mean **	Mean **
EB741	700	4	102.26	4	0.84 (0.06)	1.94 (0.69)	1.69 (0.33)
EB700	700	0.5	486.85	3	1.88 (0.05)	8.26 (2.23)	4.39 (0.52)
EB733	600	1	119.43	4	0.89 (0.16)	1.65 (0.70)	1.61 (0.51)

** Numbers in parentheses represent 1σ values.

**Table 5:** Fracture toughness of Ti_3_N_4_ samples annealed at 2 GPa. Here hardnesses and crack lengths used are from microindentation indentation experiments. A constant Young’s modulus, *E* = 500 GPa, was assumed for these calculations. Models (1) and (2) assume Palmqvist cracks, and model (3) assumes halfpenny cracks.

Sample #	T (°C)	t (h)	Number of measurements	*K_lc_*(1) MPa·m^1/2^	*K_lc_*(2)) MPa·m^1/2^	*K_lc_*(3) MPa·m^1/2^
				Mean *	Mean *	Mean *
EB741	700	4	19	2.58 (0.46)	7.09 (2.97)	3.51 (0.68)
EB746	700	2	4	2.35 (0.41)	3.99 (0.57)	3.15 (0.58)
EB747	700	1	30	2.23 (0.30)	4.64 (1.07)	3.03 (0.46)
EB700	700	0.5	35	2.79 (0.37)	7.01 (2.14)	3.84 (0.54)
EB732	600	4	3	3.55 (0.78)	10.59 (4.31)	4.91 (0.96)
EB743	600	2	26	2.26 (0.22)	4.92 (0.85)	3.11 (0.35)
EB733	600	1	6	2.56 (0.54)	5.87 (1.37)	3.47 (0.79)
EB734	600	0.5	5	2.87 (0.72)	8.84 (8.84)	3.92 (0.99)
EB744	600	0	7	2.83 (0.57)	6.29 (3.01)	3.37 (1.04)
EB699	500	3	6	2.30 (0.29)	4.82 (1.29)	3.08 (0.47)

* Numbers in parentheses represent 1σ values.

**Figure 5:**
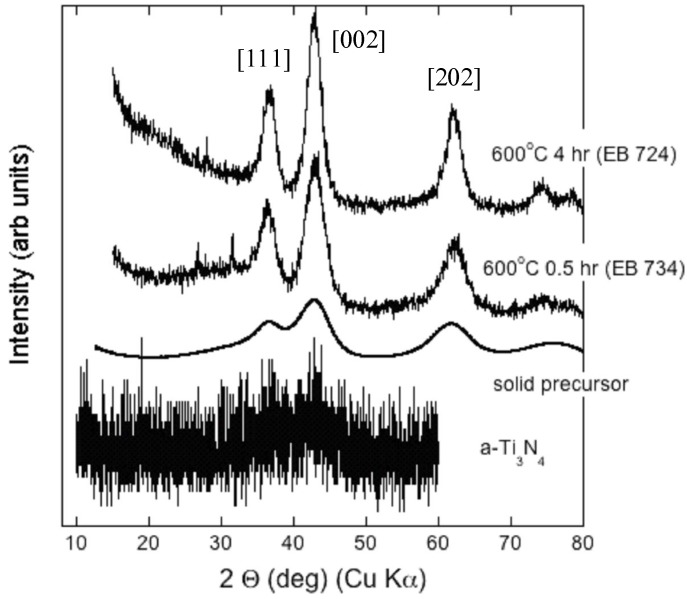
X-ray diffraction results for Ti nitride samples following high-P,T treatment compared with the amorphous powdered a-Ti_3_N_4_ starting material obtained by the chemical precursor route. The patterns for a-Ti_3_N_4_ and EB samples were obtained using laboratory instruments at Southampton University and University College London (UCL) respectively. The solid precursor pattern was obtained for a sample densified at ambient T in a diamond anvil cell at beamline I15 of the Diamond synchrotron (Chilton, UK) using an incident beam with λ = 0.442Å (A. Salamat, unpublished). All data are plotted *vs.* 2Θ values calculated for Cu K_α_ radiation.

**Figure 6:**
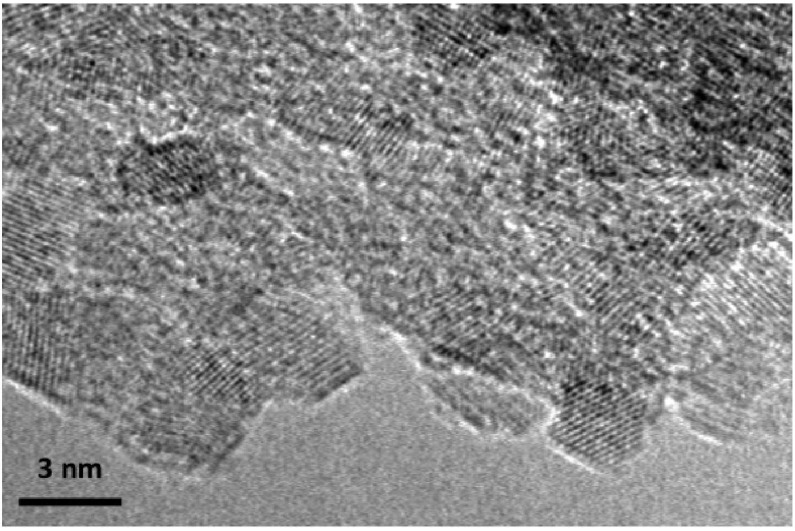
TEM micrograph of sample EB734 treated at 600 °C, 2 GPa for 0.5 h. It shows lattice fringes from crystalline grains with the *fcc* cubic rocksalt TiN_x_ structure. The particles are ~3 nm in diameter and are surrounded by amorphous material. Bulk chemical analysis indicates the composition is close to Ti_3_N_4_.

We examined the amorphous/nanocrystalline Ti_3_N_4_ samples using powder X-ray diffraction combined with TEM imaging and diffraction (Figure 5 and Figure 6). The amorphous precipitate from the metal-organic synthesis route showed a weak broad feature centered near 42° 2Θ, close to the position for the main peak of crystalline TiN (Figure 5). Following annealing at 300–450 °C in NH_3_, nanocrystalline TiN_x_ peaks emerged. Scherrer analysis indicated the nanoparticles had a mean diameter of around 2–3 nm. Following annealing at 2 GPa and 600–700 °C, the crystalline peaks sharpened slightly indicating a coarsening of the nanoparticles, resulting an average size near 3 nm in agreement with TEM results (Figure 6) [12]. As the annealing time and T increased there was a tendency to narrower X-ray linewidths, indicating an increase in nanocrystallite sizes as well as a slight reduction in the broad background signal due to the amorphous Ti_3_N_4_ component. TEM micrographs reveal a densely sintered material containing equiaxial and well defined nanocrystalline grains corresponding to the cubic TiN_x_ phase approximately 3 nm in dimension (Figure 6).

Our measurements lead to a discussion of the likely effects of local structure and nanoscale texture on properties within these highly nitrided Ti_x_N_y_ crystalline-amorphous materials. Crystalline δ-TiN_x_ with the rocksalt structure is generally sub-stoichiometric containing up to 25% vacancies on the anion sites, and materials have been produced that extend the composition range to between 0.4 ≤ x ≤ 1 [47]. Previous studies indicate that the stability range does not exceed x = 1 [39,48]. Our materials have a bulk composition near that of Ti_3_N_4_ (Table 1). Our previous XAS/EXAFS results led to a local structural model based on the rocksalt-structured TiN lattice, but with substantial vacancies occurring on both Ti and N sites [12]. TEM examination indicated nanocrystalline regions embedded within the amorphous matrix.

The influence of the vacancy content on the elastic properties in rocksalt-structured TiN_x_ materials has been studied experimentally and theoretically [39,48,49,50]. The bulk modulus achieves its highest value near 325 GPa in the Ti-N system for materials with x ≈ 1.0, and this drops to *K_o_* near 150 GPa for anion deficient compounds. Similarly, the calculated Voigt elastic stiffness drops to nearly half its initial value at for a vacancy concentration around 20% [49]. However, studies of metal carbonitrides as well as Fe-Al alloys indicates that the presence of crystalline defects can either decrease or increase hardness, due to the interplay between local mechanical softening and mechanisms involving absorption of crack propagation energy at vacancy sites [49,51]. Our results indicate that, in the case of our Ti_3_N_4_ ceramics, the softening mechanism wins. It will be interesting to see if this extends to carbonitride structures that can be prepared by a similar chemical precursor route [10,12], and also as the nanoscale crystallites evolve, with further annealing and potential N_2_ loss, to approach the stochiometric TiN composition. Another interesting direction would involve controlled incorporation of oxygen in the crystalline or amorphous component of the nanostructures that also affects the hardness [52,53,54].

In our initial discussion of the indentations produced by the microhardness measurements (Figure 1) we suggested an interesting and apparently contradictory observation of ductile *vs.* brittle behavior for the Ti_3_N_4_ nanocomposite ceramics. Usually ductile materials are hardened by creating smaller grained microstructures so that the grain boundaries interrupt the glide of dislocations under the action of shear forces. However, when the grains are too small, a transition to a different flow regime occurs as the grains slide past one another. It is feasible that such a transition from quasi-ductile to more brittle behavior could occur among the nitride ceramics with high P,T annealing that simultaneously involves reducing the amorphous content and allowing crystalline regions to grow or become more numerous within the sample. Grain slide could potentially even lead to superplastic behavior in such ultra fine grained structures [55].

## 4. Conclusions

We have examined the mechanical properties of nanocrystalline-amorphous Ti_x_N_y_ ceramics with composition close to the Ti_3_N_4_ stoichiometry produced by chemical precursor synthesis followed by high-P,T annealing. The metal nitrides do not possess high hardness but have other interesting mechanical properties that are likely due to the presence of vacancies on the metal and anion sites in both the crystalline and amorphous components of the nanocomposite ceramics. The Young’s modulus is typical of that obtained for TiN_x_ materials with compositions near x~1 and the fracture toughness is comparable with that for other important ceramics including SiC, Si_3_N_4_ and Al_2_O_3_. Interestingly, the materials seem to show apparently contradictory ductile *vs.* brittle behavior that might be developed in future studies to investigate the occurrence and applications of grain boundary sliding processes in these nitride ceramics.
